# IDO1 Activity Predicts Lung Toxicity in Patients with Unresectable Stage III NSCLC and Chemoradiotherapy

**DOI:** 10.1155/2023/3591758

**Published:** 2023-02-14

**Authors:** Linfang Wu, Yibo Gao, Daquan Wang, Yanhua Chen, Mingmin Qian, Xin Xu, Tao Zhang, Nan Bi, Luhua Wang

**Affiliations:** ^1^Department of Radiation Oncology, National Cancer Center/National Clinical Research Center for Cancer/Cancer Hospital, Chinese Academy of Medical Sciences and Peking Union Medical College, Beijing 100021, China; ^2^Central Laboratory, National Cancer Center/National Clinical Research Center for Cancer/Cancer Hospital and Shenzhen Hospital, Chinese Academy of Medical Sciences and Peking Union Medical College, Shenzhen 518116, China; ^3^Laboratory of Translational Medicine, National Cancer Center/National Clinical Research Center for Cancer/Cancer Hospital, Chinese Academy of Medical Sciences and Peking Union Medical College, Beijing 100021, China; ^4^State Key Laboratory of Molecular Oncology, National Cancer Center/National Clinical Research Center for Cancer/Cancer Hospital, Chinese Academy of Medical Sciences and Peking Union Medical College, Beijing 100021, China; ^5^Department of Thoracic Surgery, National Cancer Center/National Clinical Research Center for Cancer/Cancer Hospital, Chinese Academy of Medical Sciences and Peking Union Medical College, Beijing 100021, China; ^6^Key Laboratory of Mass Spectrometry Imaging and Metabolomics, Minzu University of China, National Ethnic Affairs Commission, Beijing 100081, China; ^7^Department of Radiation Oncology, National Cancer Center/National Clinical Research Center for Cancer/Cancer Hospital and Shenzhen Hospital, Chinese Academy of Medical Sciences and Peking Union Medical College, Shenzhen 518116, China

## Abstract

**Objectives:**

Indoleamine 2,3-dioxygenase 1 (IDO1) acts as the key rate-limiting enzyme that converts tryptophan (Trp) to kynurenine (Kyn). Its activity was primarily induced by interferon-*γ* (IFN-*γ*), which was reported to play a role in the development of acute radiation-induced pneumonitis. In this study, we aimed to investigate the correlation between IDO1 activity and radiation-induced lung toxicity (RILT) in stage III nonsmall cell lung cancer (NSCLC) patients who were treated with chemoradiotherapy (CRT).

**Materials and Methods:**

Systemic IDO1 activity was reflected by Kyn : Trp ratio. Plasma levels of Kyn and Trp in 113 stage III NSCLC patients were measured by high-performance liquid chromatography (HPLC) before the initiation of radiotherapy. Dynamic change of IDO1 activity was followed in 23 patients before, during, and after radiotherapy. We also used RNA sequencing (RNA-seq) data from the Cancer Genome Atlas Program (TCGA) database and performed gene set enrichment analysis (GSEA) to explore how IDO1 was involved in the development of RILT.

**Results:**

9.7% (11/113) of the whole group developed *G*3+ (greater than or equal to Grade 3) RILT. Preradiation IDO1 activity was significantly higher in patients who developed *G*3 + RILT than in non*G*3 + RILT patients. (*P* = 0.029, AUC = 0.70). Univariate and multivariate analyses showed that high IDO1 activity was independently associated with the risk of *G*3 + RILT (*P* = 0.034). A predictive model combining both IDO1 activity and FEV1 was established for severe RILT and displayed a moderate predictive value (AUC = 0.83, *P* < 0.001). The incidence of *G*3 + RILT was 2.6% (1/38) in patients with an IDO activity ≤0.069 and FEV1 > 59.4%, and 50.0% (6/12) in those with an IDO activity >0.069 and FEV1 ≤ 59.4%. Of 23 patients with dynamic tracking, the IDO1 activity of postradiation was significantly lower than midradiation (*P* = 0.021), though no significant differences among the three time points were observed (*P* = 0.070). Bioinformatic analysis using RNA-seq data from 1014 NSCLC patients revealed that IDO mainly functioned in the inflammatory response instead of the late fibrosis process in NSCLC patients.

**Conclusion:**

High baseline IDO1 activity combined with unfavorable baseline FEV1 was predictive of severe RILT in unresectable stage III NSCLC patients. IDO1 might play a role in the acute inflammatory response. Finding effective interventions to alleviate RILT using IDO inhibitors is warranted in the future.

## 1. Introduction

Lung cancer is one of the most lethal cancer types in China, with 85% of the patients being nonsmall cell lung cancer (NSCLC) [1, 2]. Definitive chemoradiotherapy (CRT) with consolidated immunotherapy is the standard of care for unresectable stage NSCLC patients [3]. Thoracic radiation therapy (RT) plays a pivotal role in treating NSCLC, whereas causing radiation-induced lung toxicity (RILT). With the symptoms of cough, dyspnea, fever, and fibrotic changes on computed tomography (CT), RILT can impair the lung function of patients, leading to respiratory failure and even treatment-related mortality [4, 5]. *G*3+ (greater than or equal to Grade 3) RILT or severe RILT happens in about 10% of patients and has raised increasing attention due to its high lethality [6, 7]. Thus, it is of great significance to predict RILT, particularly severe RILT, in advance of radiation. Despite emerging attempts to build predictive models, there is still no effective model available for RILT in clinical practice [6, 8–11].

Indoleamine 2,3-dioxygenase 1 (IDO1) is the key rate-limiting enzyme in the tryptophan (Trp) metabolic reaction, serving as the most active enzyme that converts the essential amino acid L-Trp into L-kynurenine (Kyn) [12]. IDO1 is highly and constitutively expressed in NSCLC patients and acts as an immune checkpoint induced by potent mediators such as interferon-*γ* (IFN-*γ*), transforming growth factor-*β* (TGF-*β*), and other proinflammatory signals in the tumor microenvironment [13–16]. A study performed in colorectal cancer cell lines and animal models has shown that IDO1 blockade protected the normal small intestinal epithelium from radiation toxicity and accelerated recovery from radiation-induced side effects [17]. Even though the mechanism was not specifically depicted in the study, it is noted that a correlation might exist between IDO status and radiation-induced toxicity.

There has been limited study relating to IDO's role in the mechanism of RILT. A review on the crosstalk among signaling pathways in RILT and immunotherapy-related lung injury (IRLI) demonstrated that cell damage caused by radiotherapy contributed to the release of numerous cytokines, including IFN-*γ*, TGF-*β*, and interleukin (IL)-6, which consequently induced lung injury [18]. Therefore, cytokines predictive of RILT were widely explored. Aso et al. reported that pretreatment IFN-*γ* was overexpressed in patients with severe radiation pneumonitis (RP) [19]. Another research demonstrated that IFN-*γ* serum levels 3 weeks after RT initiation could identify NSCLC patients predisposed to severe RP [20]. Despite the small sample size of the two studies, IFN-*γ* could be identified as an indicator for acute RP [18, 21]. TGF-*β* is another classical candidate; however, some studies failed to find the independent predictive value of TGF-*β*1 for RILT, mainly because of improper sample handling, indicating it is a less practical biomarker for RILT in the clinical setting [22, 23]. Other cytokines related to lung injury, such as tumor necrosis factor (TNF), interleukin (IL)-1, and IL-6, were not reliable either because some of the elevations happened only after RT [8]. Therefore, it would be preferable to determine a novel and stable biomarker indicating the risk of RILT before the initiation of RT in a larger cohort. IDO1 was broadly activated through the canonical IFN-*γ*-IDO axis, and IDO1 can function as a signaling molecule in the regulatory circuit in response to TGF-*β*-driven homeostatic tolerance [24, 25]. Therefore, the upregulated activity of IDO1 by proinflammatory cytokines might indicate a higher risk of developing RILT. The Kyn : Trp ratio in serum of lung cancer patients is widely used to reflect the activity of IDO1 with minimal invasiveness [14]. We measured the baseline and dynamic levels of Kyn and Trp in CRT-received NSCLC patients to explore the association between IDO1 activity and RILT. We also performed bioinformatic analyses to elucidate how IDO1 participated in the phase of RILT development.

## 2. Materials and Methods

### 2.1. Study Population and Treatment

Eligible subjects include patients pathologically diagnosed with unresectable stage III NSCLC as per the American Joint Committee on Cancer (AJCC) 8th edition cancer staging manual between January 2013 and December 2017 at our institution. All patients underwent radiotherapy with or without concurrent or sequential chemotherapy. Radiation was delivered using intensity-modulated radiotherapy (IMRT), with 6-MV X-ray implemented. The median total dose is 60 Gy (28–67 Gy) in 30 (13–33) fractions. The chemotherapy regimen mainly consisted of etoposide/cisplatin and paclitaxel/carboplatin. The study was approved by the institutional review board of the National Cancer Center, Chinese Academy of Medical Sciences, and Peking Union Medical College (IRB No. NCC-000302). All patients provided written informed consent before therapy.

### 2.2. Toxicity Evaluation

RILT, including radiation pneumonitis and clinical fibrosis, is a diagnosis of exclusion. Chest computed tomography (CT) manifestations, physical examination, and clinical symptoms were taken into account when evaluating and grading RILT according to the Common Terminology Criteria for Adverse Events (CTCAE), version 4.0. Pneumonitis caused by infectious or cardiopulmonary diseases was excluded. During CRT or follow-up period, enhanced chest CT scans were routinely carried out to evaluate lung toxicity. Respiratory symptoms of patients were also inquired about routinely. Patients who presented with cough, dyspnea, or any other respiratory symptoms would get extra CT scans and laboratory tests depending on the senior clinician's decisions.

### 2.3. Sample Collection and Measurement of Trp and Kyn

Plasma samples were prospectively collected one week before RT (pre-RT), four weeks during RT (mid-RT), and within one week after RT (post-RT). A total of 113 patients had pre-RT samples, and 23 of them had dynamic tracing at the three-time points. Liquid chromatography-tandem mass spectrometry (LC-MS/MS) was performed to quantify the plasma Trp and Kyn. The plasma samples were stored at −80°C until analysis. Eighty microliters of plasma samples were vortex mixed with 240 *μ*L of frozen acetonitrile and 8 *μ*L of internal standard solution and centrifuged at 10,000 rpm for 5 min. After centrifugation, the upper layer was concentrated and redissolved in 80 *μ*L of 2% acetonitrile solution. After filtering through a 96-well plate, five microliters of the solution was injected for LC-MS/MS analysis. The LC-MS/MS was performed on high-performance liquid chromatography coupled to a tandem mass spectrometry system (QTRAP 6500, AB SCIEX, USA) with an electrospray ionization (ESI) source and controlled by the Analyst 1.6.1 Software. The chromatographic separation was achieved on a reversed-phase Waters HSS *T*3 column (2.1 × 100 mm, 1.8 *μ*m), and the column temperature was maintained at 35°C. It was composed of water containing 0.1% formic acid (*A*) and 100% acetonitrile (*B*) using an elution gradient. The flow rate is 250 *μ*L/min. Data were acquired in the positive ion mode of the multiple reaction monitoring (MRM) scans. Raw data were first processed with Multiquant 2.2 software (AB SCIEX, USA) and then calibrated using the Norm ISWSVR program in Python 3.6.

### 2.4. Follow-up and Statistical Analyses

The median follow-up time was 63.0 months. Patients were evaluated weekly during RT, one month after RT, and then every 3 months for 2 years and every 6 months for another 3 years. Blood tests, chest and abdomen CT scans (enhanced required if without contradictions), bone scans, and brain magnetic resonance imaging (MRI) were routinely performed during the follow-up. Patients and treatment characteristics, including age, gender, Eastern Cooperative Oncology Group Performance Status Scale (ECOG-PS), smoking status, pulmonary function tests (PETs), histology, clinical stage, tumor location, dose-volume parameters such as mean lung dose (MLD) and the percentage of lung volume minus gross tumor volume receiving over 5 Gy (V5) or over 20 Gy (V20), were retrieved in the electronic medical record.

The primary endpoint was *G*3 + RILT. Mann–Whitney *U* test, Kruskal–Wallis test, paired *T*-test, and Friedman's test were adopted for general data comparison between unpaired or paired groups. The area under the curve (AUC) determined by receiver operating characteristic (ROC) analysis was employed to evaluate the predictive ability of covariates for *G*3 + RILT. Logistic regression models were used for univariate and multivariate analyses to identify the risk factor (s) of *G*3 + RILT, and median values were chosen to be the cutoff points for all continuous variables. RNA-sequencing expression profiles and corresponding clinical information for NSCLC were downloaded from the TCGA dataset (https://portal.gdc.com). R software GSVA package was used to analyze, choosing parameter as method = “ssgsea” [26]. The correlation between gene and pathway scores was analyzed by Spearman correlation. All analysis methods and R packages were implemented by R version 4.0.3. All *P* values are two-sided, and *P* < 0.05 was considered to indicate statistical significance.

## 3. Results

### 3.1. Patient Characteristics

As it is shown in Table 1, 113 NSCLC patients with qualified plasma samples were enrolled in the study. Of these, 96 (85.0%) received pulmonary function tests (PETs), and 111 (98.2%) had complete dosimetric parameters retrieved. The median age of the population was 62 years old (range, 35–80), and most (86.7%) were males. Only 16 (14.2%) of the group underwent radiotherapy alone, and 54 (47.8%) received concurrent chemoradiotherapy (CCRT). The median radiation dose was 60 Gy, with the majority (94.7%) dosed over 50 Gy.

### 3.2. Incidence of RILT

Among the 113 patients, 23.0% (26/113) developed *G*2 + RILT, 9.7% (11/113) had *G*3 + RILT, with two patients who died from fatal lung toxicity. No patients experienced Grade 4 RILT. Patients with severe lung toxicity were all identified within one year since their first irradiation during the follow-up.

### 3.3. Correlation between IDO Activity and RILT

**Figure 1:**
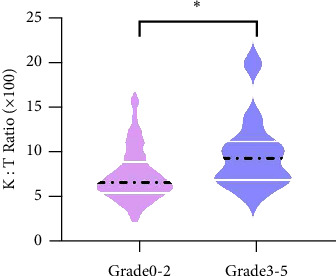
The comparison of preradiation IDO activity between patients with *G*3 + RILT and non *G*3 + RILT (*P*=0.029). *K* : *T* ratio is an indicator of IDO activity.

The median Kyn : Trp ratio of the whole group was 0.07 before radiotherapy. Kyn : Trp ratio indicates the level of IDO activity as previously described. The median preRT Kyn : Trp ratio was 0.09 in patients with *G*3 + RILT, which was significantly higher than in the non-*G*3 + population (0.09 vs. 0.06, *P*=0.029, Figure 1).

The analyses of risk factors for *G*3 + RILT are shown in Table 2. Continuous covariates including forced expiratory volume in the first second (FEV1), forced vital capacity (FVC), diffusing capacity for carbon monoxide (DLCO), V5, V20, MLD, Trp, Kyn, and Kyn : Trp ratio were dichotomized using the median value as the cutoff point. Three risk factors with a *P* value less than 0.1 were identified in the univariate analysis and then included in the multivariate analysis. The result showed that the preRT Kyn : Trp ratio was significantly correlated with the rate of *G*3 + RILT (OR: 10.21; 95% confidence interval [CI]: 1.20–87.30; *P*=0.034). High baseline FEV1 tended to be a protective factor for lung toxicity, although no significance was noted (OR: 0.21; 95% CI: 0.40–1.13; *P*=0.070).

**Figure 2:**
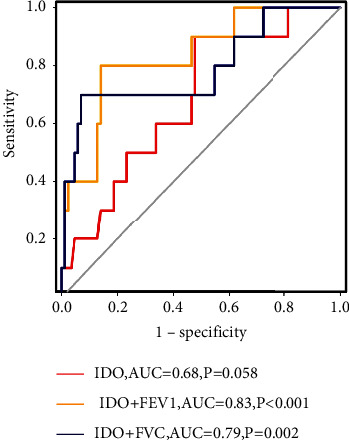
The combination of IDO activity with FEV1 or FVC showed improvement in the prediction of *G*3 + RILT as compared with IDO activity alone. Abbreviations: FVC, forced vital capacity; FEV1, forced expiratory volume in the first second.

Then ROC analysis was performed on 96 patients to explore the combined predictive value of IDO activity and PETs parameters for *G*3 + RILT. Three ROC curves are shown in Figure 2. The combination of IDO activity and FEV1 displayed the best predictive ability (AUC = 0.83, *P* < 0.001), as compared with IDO and FVC (AUC = 0.79, *P*=0.002), or IDO activity alone (AUC = 0.68, *P*=0.058). By using ROC analysis, the optimal cutoff points for FEV1 and IDO activity were calculated as 59.4% and 0.069, respectively. The incidence of *G*3 + RILT was 2.6% (1/38) in patients with an IDO activity ≤0.069 and FEV1 > 59.4%, and 50.0% (6/12) in those with an IDO activity >0.069 and FEV1 ≤ 59.4%.

### 3.4. Dynamics of IDO Activity

**Figure 3:**
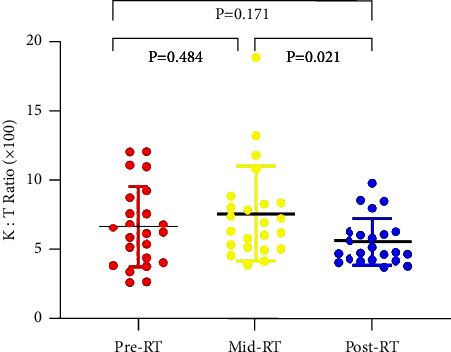
The levels of IDO activity at three time points during radiotherapy (before the initiation of RT, two weeks after the initiation of RT, and within one week after RT.

IDO activity was dynamically monitored in 23 patients during RT. The median Kyn : Trp ratio (*∗*100) was 6.22 before RT, 7.02 at four weeks after RT, and 4.86 after RT, respectively. According to the Friedman test, the IDO activity was not significantly different among the three time points (*P*=0.07). Paired comparisons are shown in Figure 3. IDO levels descended prominently at the end of RT as compared to mid-RT levels (*P*=0.021) but remained relatively stable in the first four weeks of RT.

### 3.5. Correlations between IDO1 and Six Pathways

**Figure 4:**
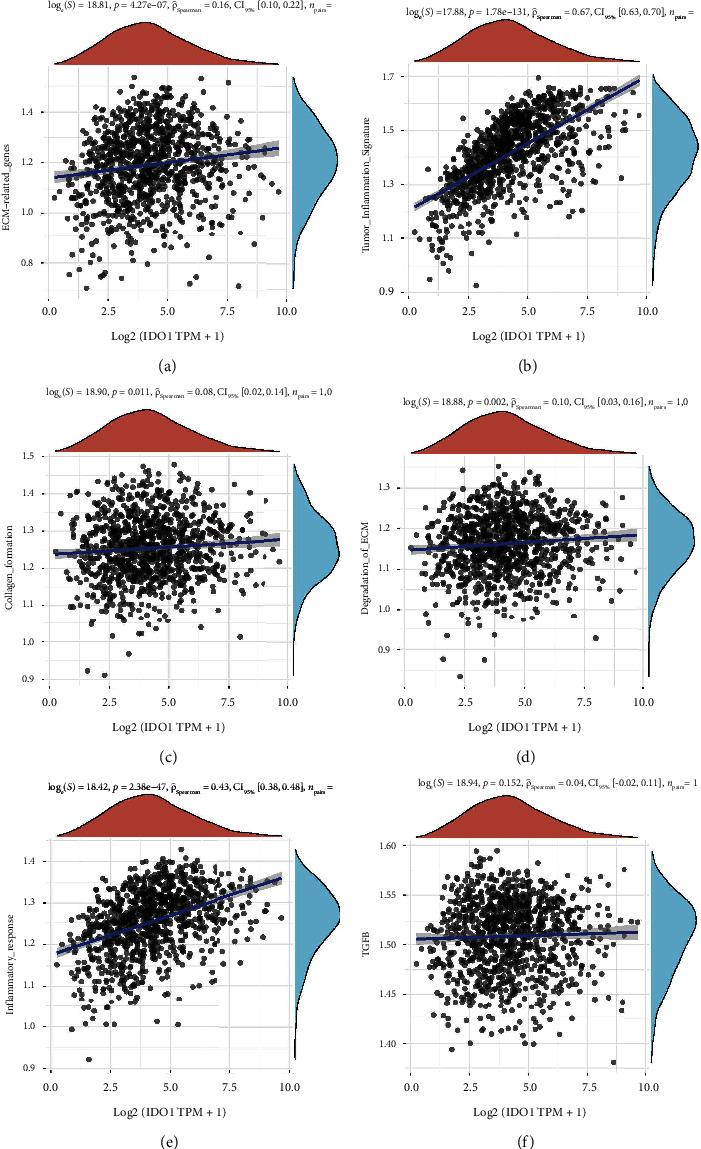
The correlation between IDO1 and pathway score was analyzed with Spearman. The abscissa represents the distribution of the gene expression, and the ordinate represents the distribution of the pathway score. The density curve on the right represents the trend in the distribution of pathway immune score, the upper density curve represents the trend in the distribution of the gene expression. The value on the top represents the correlation *P* value, correlation coefficient, and correlation calculation method. Six pathways are included: (a) ECM-related genes; (b) tumor inflammation signature; (c) collagen formation; (d) degradation of ECM; (e) inflammatory response; (f) TGFB.

To explore how IDO1 took part in the development of RILT, we conducted a correlation analysis between IDO1 and six common gene pathways involved in pulmonary fibrosis and inflammation based on the TCGA database. The six pathways related to genes were involved in extracellular matrix (ECM), collagen formation, degradation of ECM, TGF-*β*, tumor inflammation, and inflammatory response. As it is shown in Figure 4, IDO1 expression was significantly correlated with the inflammatory response in NSCLC patients (Spearman correlation score = 0.67, 95% CI: 0.63–0.70, *P*=1.78*e*–131), while the correlation scores of the other five pathways were too low to indicate any correlation. By the fact that all severe RILT in our cohort occurred within six months from the start of RT, during which the acute or early phase of RILT usually happened, we assume that IDO1 was primarily correlated with inflammatory response rather than fibrosis [27].

## 4. Discussion

Data from our study demonstrate that the combination of pre-RT IDO activity and FEV1 can be used to construct a predictive model for severe RILT. To our knowledge, this is the first study in the literature that associates this novel metabolomics biomarker with radiation-induced toxicity in lung cancer patients. We also use a bioinformatic tool to establish a correlation between IDO1 and acute inflammatory response and thus enlighten thinking in the usage of IDO inhibitors to alleviate RILT. More importantly, both IDO1 activity and FEV1 were obtained relatively easily before the initiation of radiation, indicating that the model can serve as a promising and convenient tool for prescribing individualized RT plans in clinical practice.

IDO1 could be used as a blood biomarker for *G*3 + RILT. The rationale might be that IDO1 was primarily induced by IFN-*γ* and served as a responder to TGF-*β*, both of which were involved in inducing lung injury and predictive of RILT. Therefore, high levels of pre-RT IDO1 might predispose patients to acute RP. This hypothesis was per the analysis results from the TCGA database. It is worth noting that even though we tracked 23 patients dynamically and observed no significant variation in IDO1 activity during RT, we could not assess whether the levels of IDO activity at mid-RT or post-RT were predictive of RILT due to the small sample size. Further research is needed to validate our results in a larger population, and the internal mechanisms remain to be explored.

Our study might enlighten a more extensive usage of IDO1 inhibitors to reduce radiation-induced toxicity in the future. Much attention of IDO1 in RT was its role as a targetable immune mediator, without mentioning toxicity. Research has shown that IDO1 levels were influenced heterogeneously under different RT schemes due to the extent of immune activation [28, 29]. A study reported that IDO1 blockage could overcome radiation-induced “Rebound Immune Suppression” in the tumor microenvironment and sensitized Lewis lung carcinoma (LLC) tumors to hypo-fractionated RT [30]. Another study using the LLC model also revealed that the combination of IDO inhibitor 1-methyltryptophan (1-MT) and 10 Gy RT therapy was more effective than either treatment alone [31]. Intriguingly, Chen et al. found out that IDO1 blockade could protect the normal small intestinal epithelium from radiation toxicity in preclinical models [17]. Even though the relevant mechanism was unclear, the study shed light on related explorations, and it is promising to develop a more extensive usage of IDO1 inhibitors to increase efficacy and simultaneously decrease radiation-related toxicity in the future.

The consensus on whether PETs parameters were predictive of RILT has not been reached yet. Wang et al. [11] reported poor baseline pulmonary function did not increase the risk of symptomatic RILT in 260 NSCLC patients treated with CRT. On the contrary, Zhou et al. [32] concluded that a combination of DLCO% and MLD could predict the risk for severe RP among patients with pretreatment moderate pulmonary dysfunction. A multicenter study demonstrated that FEV1, DLCO, and FeNO before CRT predict the development of *G*2 + RP [33]. Our study also showed that the addition of FEV1 could significantly increase the model's predictive ability for *G*3 + RILT. Besides, PETs are widely used in clinical practice with easily accessible data, so it is convenient to include PETs parameters in predictive models.

Our study has several limitations. Firstly, this study was performed in a single center, and only a small proportion of the participants' plasma was longitudinally followed. These results require further validation in a larger population among multiple centers. Secondly, this study only analyzed clinical characteristics, dosimetric factors, pulmonary function parameters, and metabolic data. A more comprehensive model of RILT incorporating genetic profiles and radiomics features warrants to be developed. Finally, since no patients in our cohort ever received immunotherapy, whether IDO1 activity could display similar predictive ability after consolidated immunotherapy is still unknown.

In conclusion, this study demonstrated that high baseline IDO1 activity combined with unfavorable baseline PETs was predictive of severe RILT in unresectable stage III NSCLC patients. IDO might mainly function in the early inflammatory phase of RILT development instead of the late fibrosis process. Finding effective interventions to alleviate RILT using IDO inhibitors is warranted in the future.

## Figures and Tables

**Table 1:** Patient baseline characteristics.

Variables	No. of patients	Data
Sex (male/female)	113	98/15
Age	113	62 (35–80)
ECOG-PS (<2 vs. ≥2)	113	63/50
Smoking history (yes/no)	113	92/21
Clinical stage (IIIA/IIIB/IIIC)	113	26/64/23
Histology (SCC vs. non-SCC)	113	76/37
Location (lower vs. other)	113	34/79
Therapy (CCRT/SCRT/RT alone)	113	54/43/16
FVC%	96	74.00 (36.30–109.60)
FEV1%	96	73.20 (18.70–113.50)
DLCO%	96	63.70 (12.10–122.00)
Radiation dose (Gy)	113	60.00 (27.90–67.00)
*V*5%	113	55.05 (31.65–88.49)
*V*20%	113	23.69 (12.00–46.80)
MLD (Gy)	113	14.36 (7.84–22.01)
Tryptophan (*μ*mol/L)	113	26.70 (11.44–40.03)
Kynurenine (*μ*mol/L)	113	1.79 (0.56–5.03)
Kyn/Trp ratio (× 100)	113	6.87 (2.58–19.82)

All continuous variables in the dataset present with median and range values. ECOG-PS, eastern cooperative oncology group-performance status; SCC, squamous cell carcinoma; CCRT, concurrent chemoradiotherapy; SCRT, sequential chemoradiotherapy; RT, radiotherapy; FVC, forced vital capacity; DLCO diffusing capacity for carbon monoxide; FEV1, forced expiratory volume in the first second; *V*5, percentage of lung volume minus gross tumor volume receiving >5 Gy; *V*20, the percentage of lung volume minus gross tumor volume receiving >20 Gy; MLD, mean lung dose; Kyn/Trp ratio, an indicator of IDO activity.

**Table 2:** Univariate and multivariate analyses of severe RILT (grade ≥3) in patients with stage III non-small-cell lung cancer treated with chemoradiotherapy (*N* = 113).

Variable	Univariate analysis			Multivariate analysis		
	Odds ratio	95% CI	*p*-value	Odds ratio	95% CI	*p*-value
Age (≥62 vs. <62)	3.52	0.88–14.02	0.075	2.57	0.57–11.56	0.218
ECOG-PS (2 vs. 1)	1.58	0.45–5.52	0.472			
Tumor location (lower lobe vs. nonlower lobe)	1.23	0.44–3.47	0.691			
Histology (squamous vs. nonsquamous)	0.43	0.09–2.08	0.291			
%FVC (*n* = 96) (≥74.0% vs. <74.0%)	0.41	0.10–1.69	0.216			
%DLCO (*n* = 96) (≥63.7% vs. <63.7%)	0.64	0.17–2.42	0.507			
%FEV1 (*n* = 96) (≥73.2% vs. <73.2%)	0.22	0.04–1.08	0.063	0.21	0.40–1.13	0.070
Concurrent radiotherapy (yes vs. no)	1.75	0.48–6.35	0.395			
*V*5% (≥55.0% vs. <55.0%)	1.82	0.50–6.60	0.362			
*V*20% (≥23.9% vs. <23.9%)	0.80	0.23–2.79	0.728			
MLD (Gy) (≥14.4 vs. <14.4)	0.80	0.23–2.79	0.728			
Tryptophan (*μ*mol/L) (≥26.7 vs. <26.7)	0.35	0.09–1.38	0.133			
Kynurenine (*μ*mol/L) (≥1.80 vs. <1.80)	1.25	0.39–4.35	0.728			
Kyn/Trp ratio (× 100) (≥6.9 vs. <6.9)	11.3	1.39–91.14	0.023	10.21	1.20–87.30	0.034

Continuous variables were dichotomized using the median as the cutoff point. ECOG-PS, eastern cooperative oncology group-performance status; SCC, squamous cell carcinoma; FVC, forced vital capacity; DLCO, diffusing capacity for carbon monoxide; FEV1, forced expiratory volume in the first second; *V*5, the percentage of lung volume minus gross tumor volume receiving >5 Gy; *V*20, the percentage of lung volume minus gross tumor volume receiving >20 Gy; MLD, mean lung dose; Kyn/Trp ratio, an indicator of IDO activity.

## Data Availability

The data presented in this study are openly available at https://www.ebi.ac.uk/metabolights/MTBLS5267, with the accession number MTBLS5267.
